# Depletion of CDC5L inhibits bladder cancer tumorigenesis

**DOI:** 10.7150/jca.32850

**Published:** 2020-01-01

**Authors:** Ziwei Zhang, Weipu Mao, Longsheng Wang, Mengnan Liu, Wentao Zhang, Yuan Wu, Junfeng Zhang, Shiyu Mao, Jiang Geng, Xudong Yao

**Affiliations:** Department of Urology, Shanghai Tenth People's Hospital, Tongji University, Shanghai 200072, P. R. China.

**Keywords:** CDC5L, bladder cancer, tumorigenesis.

## Abstract

Cell division cycle 5-like (CDC5L) protein is a cell cycle regulator of the G2/M transition and has been reported to participate in the catalytic step of pre-messenger RNA (mRNA) splicing and DNA damage repair. Recently, CDC5L was also found to act as a candidate oncogene in osteosarcoma and cervical tumours. However, the role of CDC5L expression in bladder cancer remains unclear. Here, we analysed the expression and clinical significance of CDC5L in bladder cancer tissues. The expression of CDC5L in fresh bladder cancer tissues and paraffin-embedded slices was evaluated by western blot and immunohistochemistry, respectively. We found that CDC5L was highly expressed in bladder cancer. The expression of CDC5L was significantly associated with bladder cancer pathology grade and Ki67 expression. Univariate and multivariate analyses showed that high CDC5L expression was an independent prognostic factor for the survival of bladder cancer patients. To determine whether CDC5L could regulate the proliferation of bladder cancer cells, we transfected bladder cancer cells with an interfering RNA targeting CDC5L and then investigated cell proliferation with a cell counting kit (CCK)-8, flow cytometry assays, colony formation and xenograft assay analyses. Our results indicate that knockdown of CDC5L inhibits proliferation of bladder cancer cells. In addition, reduced expression of CDC5L induced apoptosis of bladder cancer cells and inhibited their migration, invasion and EMT. These findings suggest that CDC5L might play an important role in bladder cancer and thus be a promising therapeutic target of bladder cancer.

## Introduction

Bladder cancer (BCa) is one of the most prevalent cancers of the genitourinary system and occurs more commonly in men than women 1. Nearly three-quarters of bladder cancers are superficial [stages Ta, T1, carcinoma *in situ* (CIS)] at presentation which are now termed as non-muscle invasive bladder cancer (NMIBC) 2, 3.While the clinical treatment of bladder cancer has made great progress, the prognosis of BCa patients still remains unsatisfactory due to a high incidence of metastasis and invasion 4, 5.A number of gene mutations are closely related to the development of bladder cancer 6.Therefore, research into the molecular aetiology may provide insights into the mechanism of the development of bladder cancer.

The Cell division cycle 5-like (CDC5L) protein has been well-known a significant similarity with the Schizosaccharomyces pombe cdc5 gene product, which is a cell cycle regulator essential for the G2/M transition 7-10. CDC5L interacts with the cell cycle checkpoint protein ATR and activates effectors downstream of ATR, including Chk1 and Rad17. Interference with CDC5L inactivates the S phase cell cycle checkpoint, thus increasing drug sensitivity 11. In addition to affecting the cell cycle, CDC5L protein is also a member of the spliceosome complex and is involved in pre-mRNA splicing 12-14. This suggests that CDC5L may be a very active protein. CDC5L also plays a key role in some human somatic tumours. CDC5L promotes the transcriptional activation of the hTERT promoter as an oncogene in colorectal cancer. It is reported that CDC5L possesses potential oncogenic activity in osteosarcoma and cervical tumours 15, 16. CDC5L is closely related to the mitotic stage of the cell cycle, so it is considered a potential target for tumour therapy 17, 18. At present, there is no report on the role of CDC5L in bladder cancer and related mechanisms.

In this study, we demonstrated the expression and potential function of CDC5L in bladder cancer. These results suggest that CDC5L plays a crucial role in bladder cancer and may provide a new potential target for cancer therapy targets in bladder cancer.

## Materials and methods

### Patients and tissue specimens

Surgical specimens of bladder cancer tissues and paired normal bladder tissues were obtained from the Department of Urology, Shanghai Tenth People's Hospital, Tongji University (Shanghai, China) from January 2008 to December 2018. Following surgery, fresh tissue specimens were immediately snap-frozen in liquid nitrogen and stored at -80 °C until further use. Written informed consent from all patients or their relatives and approval from the Ethics Committee of the Tenth People's Hospital was obtained.

### Tumour microarray and immunohistochemistry

Paraffin sections were taken from patients with bladder cancer for tissue microarray. Human bladder cancer and adjacent normal tissues were fixed in cold 4% paraformaldehyde. Tumour-rich areas were board-certified by pathologists. After constructing the tissue microarray, the sections were stained for CDC5L. The pathological sections were assessed separately by at least two pathologists. Five fields of view were randomly selected from bladder cancer tissues and normal bladder tissues for histological scoring. Intensity was evaluated in comparison with the control and scored as follows: 0 (no staining), 1 (weak staining = light yellow), 2 (moderate staining = yellow brown), and 3 (strong staining = brown). Scores representing the proportion of positively stained tumour were as follows: 0, <10%; 1, 11-25%; 2, 26-50%; 3, 51-75%; and 4, >75%. Scores from the two scales were combined, and we divided the expression of CDC5L into two grades: scores from 0-3 were counted as low expression, while scores from 4-7 were counted as overexpression.

### Cell lines and culture

Human bladder cancer cell lines (T24, UMUC3 and J82) and human bladder epithelial permanent cell line SV-HUC-1 were purchased from the American Type Culture Collection (ATCC, Rockville, USA). SV-HUC-1 cells were maintained in F12K medium (Sigma-Aldrich, St. Louis, MO, USA). T24 and UMUC3 cells were cultured in RPMI-1640 medium (Gibco, Rockville, MD, USA), and J82 cells were grown in Dulbecco's modified Eagle's medium (Gibco). All cell culture media were added with 10% foetal bovine serum (FBS; Gibco) and 1% penicillin/streptomycin (HyClone, Logan, UT, USA). The cell lines were incubated at 37 °C in a humidified atmosphere of 5% CO_2_.

### RNA extraction and quantitative real-time PCR

Total RNA was extracted from the cultured cells using TRIzol reagent (Invitrogen, Carlsbad, CA, USA). Purity and concentration of the RNA were determined using an ND-2000 spectrophotometer (Thermo Fisher Scientific, Inc., Carlsbad, CA, USA). The RNA was reverse-transcribed into cDNA using a cDNA synthesis kit (Takara, Kyoto, Japan), and qRT-PCR was performed using a SYBR Green PCR Kit (Takara Biotechnology, Dalian, China) with an ABI Prism 7500 Sequence Detection System (Applied Biosystems, Foster City, CA, USA). The primers for PCR analysis were as follows: CDC5L: 5′-TCTCT GAAG CTCC TCTC GGC-3′ (forward) and 5′-CATC CTCG GTAT TCCT CCATACG-3′ (reverse). β-actin: 5'-CCTGGCACCCAGCACAAT-3' (forward) and 5'-GGGCCGGACTCGTCATAC-3' (reverse). PCR conditions: 2 min at 95 °C, followed by 40 cycles of 15 sec at 95 °C and 30 sec at 60 °C. CDC5L mRNA expression was normalized to the β-actin mRNA. The relative expression levels of CDC5L were analysed using the 2^-ΔΔCt^ method. Each sample was tested in triplicate.

### Cell transfection

Three CDC5L siRNA sequences were designed by GenePharma(Shanghai, China) and finally we selected the one with the most obvious effect. The sequence of CDC5L siRNA was: 5'-CGCUGUUUGGUAUUUGGUAUU-3'. CDC5L siRNA (si-CDC5L) and negative control siRNA (si-NC) were all obtained from GenePharma. Lipofectamine 3000 (Invitrogen; Thermo Fisher Scientific, Inc., USA) was used for transfection, according to the manufacturer's instruction. The expression of CDC5L was examined at RNA and protein levels.

### Cell proliferation assay

Cell proliferation rate was measured using a Cell Counting Kit-8 (CCK-8; Dojindo, Japan), according to the manufacturer's instructions. Simply speaking, the transfected cells were plated into 96‐well plates at a density of 2x103 cells/well and grown for up to 5 days. Subsequently, CCK-8 reagent (10 μl) was added to each well, and the cells were incubated at 37 °C for 2 h. The absorbance at 450 nm was determined using a microplate spectrophotometer (BioTek Instruments Inc., Winooski, VT, USA).

### Colony formation assay

The transfected cells were seeded into 6-well plates at a density of 1,000 cells/well. After approximately 10 days from plating, the colonies were washed three times with PBS, fixed with methanol and stained with 0.1% crystal violet solution. Cell colonies ≥50 cells were counted under a light microscope (Olympus Corporation, Tokyo, Japan). The experiment was performed at least three times.

### Xenograft assays in nude mice

The lentivirus expressing the shRNA against CDC5L was produced by Lingke Biotechnology (Shanghai, China). T24 and UMUC3 cells were infected with LV-sh-CDC5L or LV-sh-NC and then selected using puromycin (Sigma-Aldrich). After generating stably transfected LV-sh-NC and LV-sh-CDC5L cell lines, cells were injected subcutaneously (3 x106 in 0.1 mL of PBS) into mice. Tumour growth curves were measured weekly with a Vernier caliper from week 1-5. The tumour volume was calculated using the following formula: tumour volume [mm3] = (length [mm]) × (width [mm])2 × 0.5. After 5 weeks, the mice were sacrificed and tumour xenografts were resected and then stored at ‐80 °C until further use. All animal studies were approved by the Institutional Animal Care and Use Committee of the Shanghai Tenth People's Hospital of Tongji University.

### Cell apoptosis assay

Annexin-V/PI apoptosis detection kit (BD Pharmingen, Franklin Lakes, NJ, USA) was used to measure cell apoptosis, according to the manufacturer's instructions. First, the cells were collected, washed twice with cold PBS, resuspended in Annexin V-FITC and propidium iodide (PI), and stained in the dark at room temperature for 15 min. Subsequently, apoptosis rates were analysed by flow cytometry (fluorescence-activated cell sorting, BD Biosciences).

### Scratch wound healing assay

For the scratch healing assay, the transfected cells were grown to full confluence in six-well plates. Then, the cells were wounded using a 200 μl sterile pipette tip, washed 3 times with PBS, and cultured in RPMI-1640 medium without FBS. The wounds were continuously photographed at a magnification of x5 using an optical system microscope at 0 and 48 hours.

### Transwell invasion assay

6.5-mm Transwell chambers with 8‐μm pore polycarbonate membrane inserts (BD Biosciences, San Jose, CA, USA) were used for invasion assays. A total of 5×10^4^ cells/Transwell were plated in the top chamber of the Transwells in 200 μl of FBS-free RPMI-1640 medium. The bottom chamber was filled with 500 μl of cell culture medium containing 10% FBS. Furthermore, the insert of each Transwell was precoated with Matrigel (BD Biosciences, Franklin Lakes, NJ, USA), and the cells were allowed to invade. After about 24 h, the cells remaining in the upper surface of the inserts were carefully wiped with a moist cotton swab, and the invading cells in the lower surface of the inserts were fixed with 70% ethanol for 15 min and stained with 0.1% crystal violet for 15 min and then observed under a light microscope (Olympus Corporation). Invasion abilities were defined as the number of invading cells. The experiments were repeated at least three times.

### Western blot assay

Total proteins of tissues were extracted using ice-cold RIPA buffer (Sigma-Aldrich) containing a protease inhibitor. The protein concentration of lysates was measured using the BCA method. An equal amount of protein was separated by 10% sodium lauryl sulfate-polyacrylamide gels (SDS-PAGE) and transferred to nitrocellulose (NC) membranes. Afterward, the membranes were blocked with 5% non-fat milk in PBS for 1 h at room temperature and immunoblotted overnight with primary antibodies at 4 °C. All primary antibodies were purchased from Abcam (Cambridge, Cambridge, UK). Then, the membranes were incubated with a fluorescence‐conjugated secondary antibody (926‐68072 or 926-32210, LI-COR Biosciences, Shanghai, China) for 1 h at room temperature. Subsequently, the membranes were washed in PBST three times and the protein bands visualized and quantified using the Odyssey two‐colour infrared laser imaging system (LI-COR Biosciences, Lincoln, NE, USA). Three independent experiments were carried out for each assay.

### Statistical analysis

SPSS 16.0 software (SPSS Inc., Chicago, IL, USA) and GraphPad Prism 7 (GraphPad Software, Inc., La Jolla, CA, USA) were used to analyse the resulting data. Chi-square tests or Fisher's exact tests were used to assess the association between clinical pathological features and CDC5L expression, and Student's t test was used for comparisons between two groups. P<0.05 was considered statistically significant.

## Results

### CDC5L is upregulated in bladder cancer cells and tissues

**Figure 1:**
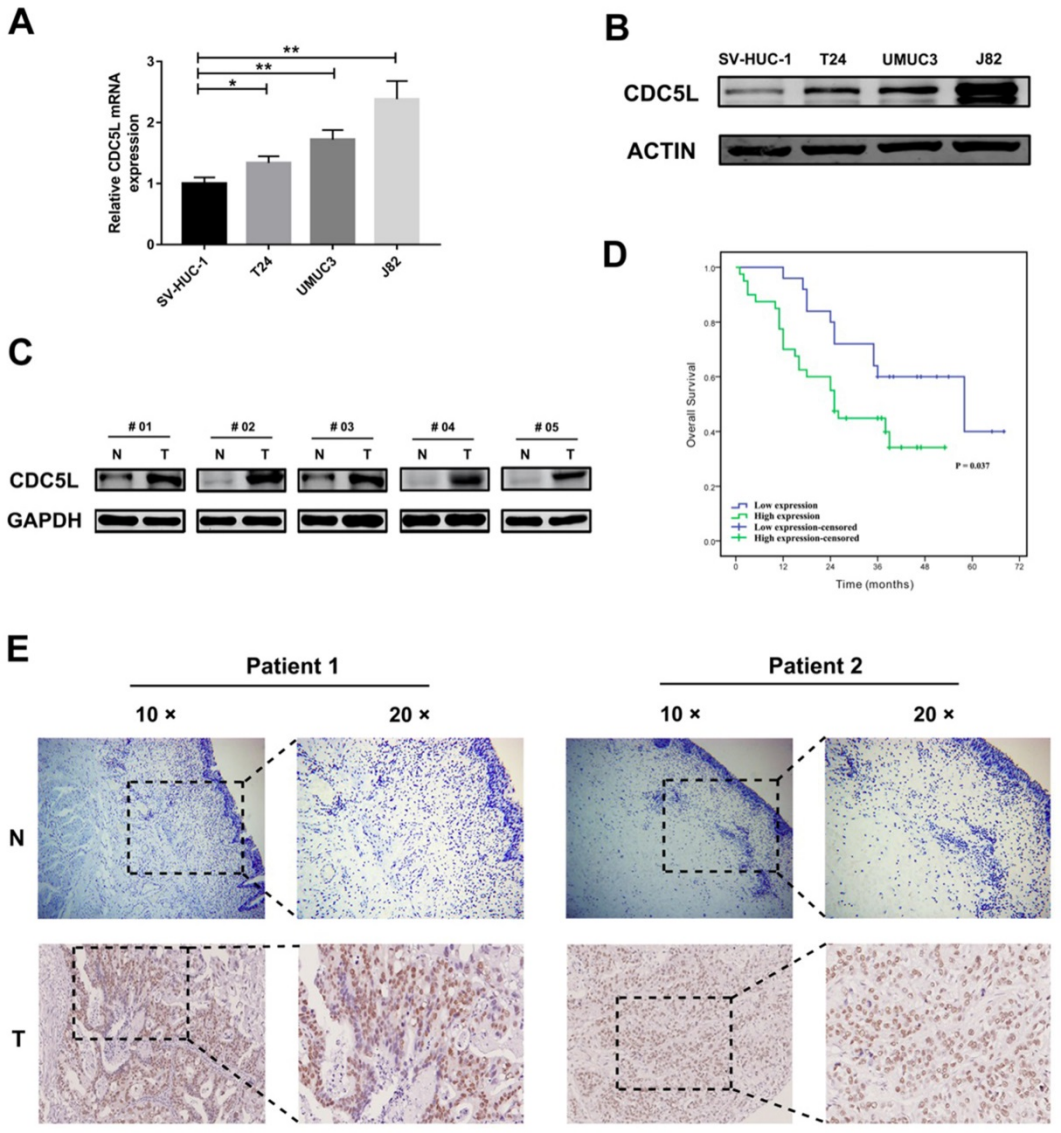
CDC5L is upregulated in human bladder cancer cell lines and tissues. Protein (A) and mRNA (B) expression of CDC5L in bladder cancer cell lines (T24, UMUC3 and J82) and the immortalized human normal bladder epithelial cell line SV-HUC-1 determined by western blot and qRT-PCR, respectively. (C) protein levels of CDC5L in bladder cancer tissues (T) and paired adjacent normal tissues (N) detected by western blot. (D) Kaplan-Meier survival analysis of patients with bladder cancer according to CDC5L expression status. Kaplan-Meier postoperative survival curve for high CDC5L expression group (40 patients) and low CDC5L expression group (25 patients). Patients in the high-expression CDC5L group had significantly shorter overall survival (P < 0.01). (E) Immunohistochemistry (IHC) staining of CDC5L in bladder cancer tissues (T), and in adjacent normal tissues (ANT). Representative images are shown. Scale bar = 200 μm for 10 × and 100 μm for 20 ×.

Previous studies have revealed that CDC5L plays an active role in the development of cancers. To determine the expression pattern of CDC5L in bladder cancer cells and tissues, western blot analysis and qPCR were performed to investigate the expression pattern of CDC5L in bladder cancer cells and tissues. The results suggest that the mRNA and protein expression of CDC5L were generally increased in the 3 bladder cell lines T24, UMUC3 and J82 compared with the human bladder epithelial permanent cell line SV-HUC-1(Fig. 1A and 1B). Consistently, CDC5L expression was also higher in bladder cancer tissues than in the matched normal bladder tissues (Fig. 1C).

We took sixty-five pairs of bladder cancer tissues and their adjacent normal tissue for tissue microarray. The stained tissue microarrays indicated that CDC5L was highly expressed in 61.5% (40/65) of bladder cancer tissues compared with 38.5% (25/65) of the matched normal tissues and the high expression of CDC5L between bladder tumour tissues and matched normal tissues was statistically significant (Fig. 1E). The clinicopathological factors of the 65 patients are listed in Table 1. As shown in Table 1, the expression of CDC5L was not related to age, gender, smoking status, BMI, diabetes status, or T stage but significantly correlated with histologic grade, N stage and M stage. Kaplan-Meier survival curves indicate that the high CDC5L expression was significantly associated with poorer overall survival and the difference was statistically significant (Fig. 1D). From these data, we infer that CDC5L might contribute to the progression of bladder cancer.

### Knockdown of CDC5L inhibited the growth of bladder cancer *in vitro* and *in vivo*

**Figure 2:**
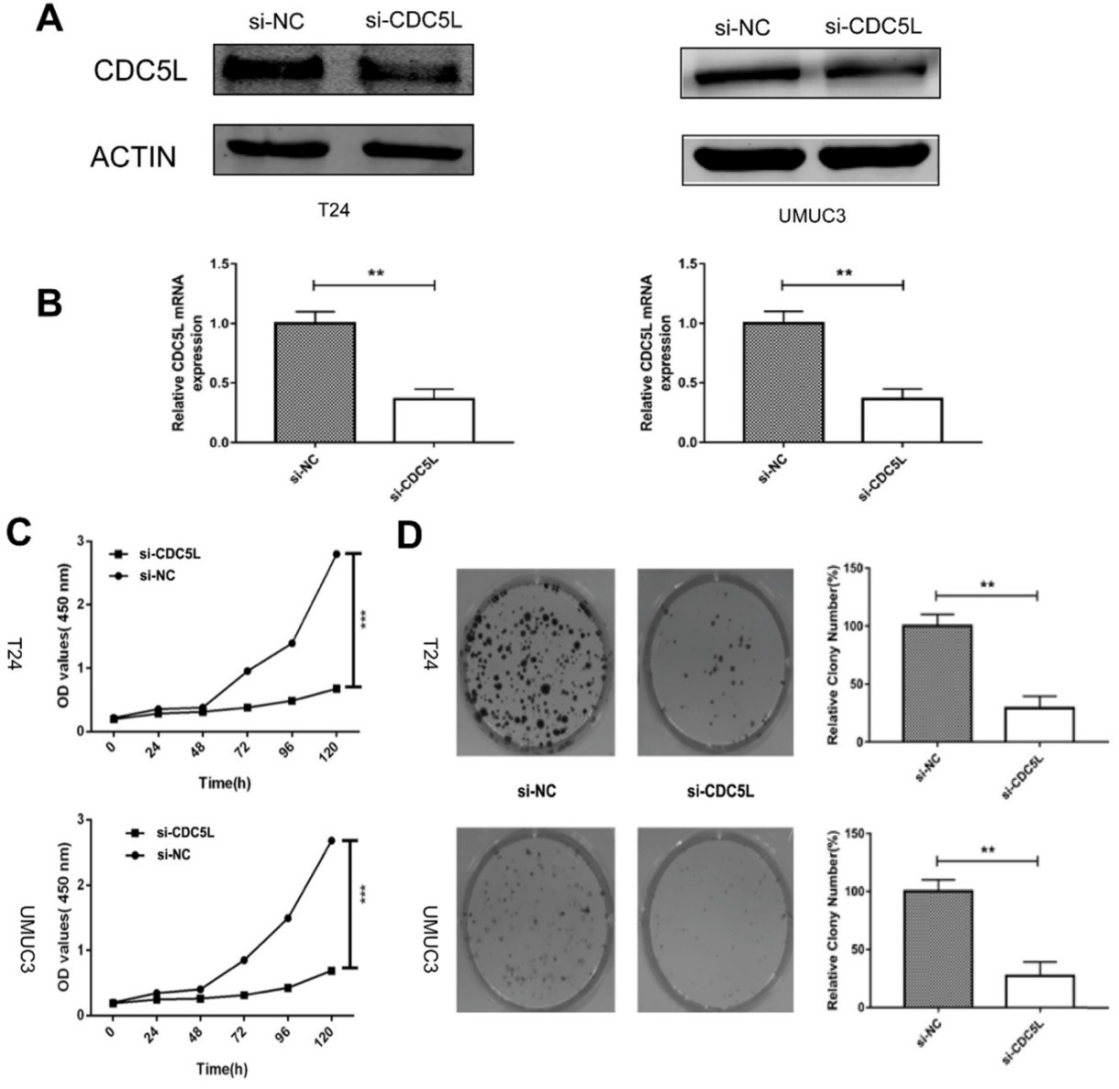
Knockdown of CDC5L inhibits cell growth of T24 and UMUC3 cells *in vitro*. (A) CDC5L protein expression was determined by western blot 72 h after transfection of si-CDC5L or si-NC. (B) CDC5L mRNA expression was detected by qRT-PCR 48 h after transfection of si-CDC5L or si-NC. CCK8 assay (C) and colony formation assay (D) were used to determine proliferation and colony-forming ability of si-CDC5L or si-NC-transfected T24 and UMUC3 cells. **P<0.01vs. si-NC groups (n=3).

To determine the biological effects of CDC5L on bladder cancer cell growth, T24 cells and UMUC3 cells were transfected with si-CDC5L or si-NC, respectively. The result revealed that the si-CDC5L was successfully transfected into the cells (Fig. 2A and 2B). Subsequently, CCK-8 and colony formation assays were used to study cell proliferation. The CCK-8 proliferation assay indicated that the proliferation of T24 and UMUC3 cells was suppressed markedly following infection with si-CDC5L (Fig. 2C). The colony formation assay revealed that knockdown of CDC5L significantly inhibited the proliferation of T24 cells and UMUC3 cells, when compared with the respective control (Fig. 2D).

**Figure 3:**
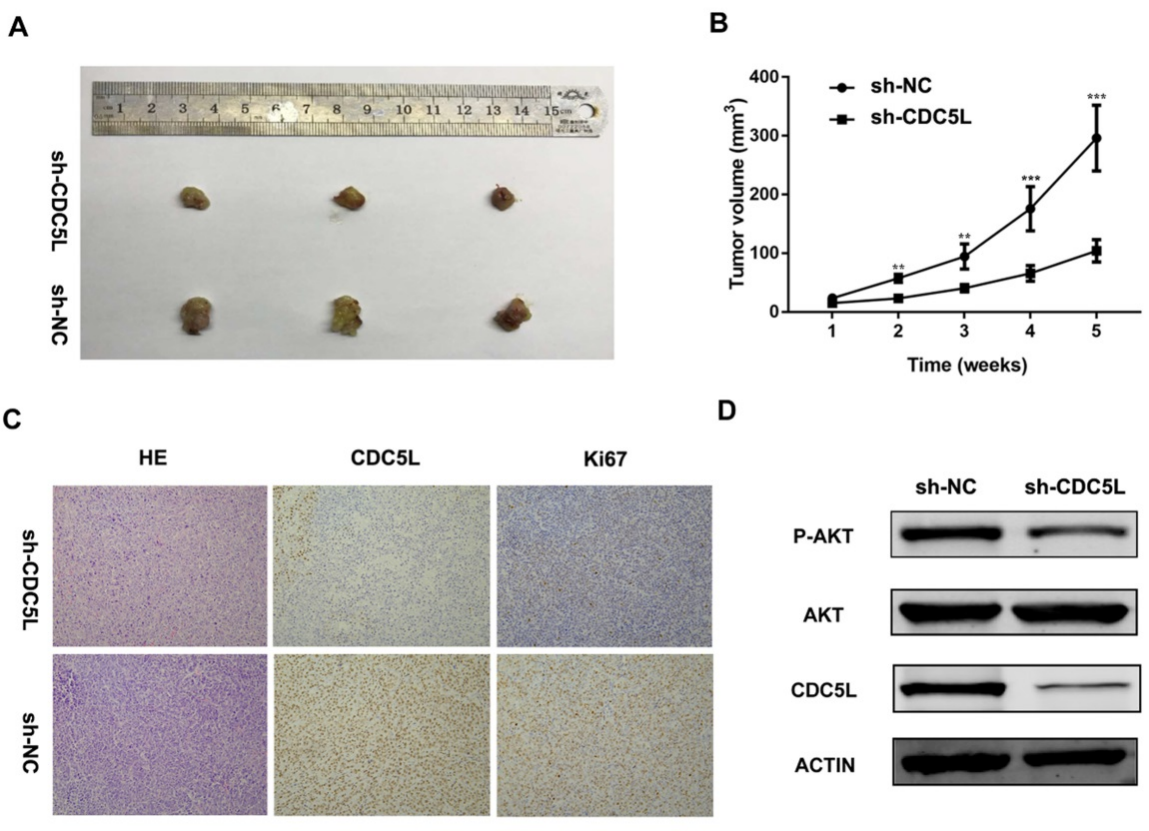
Knockdown of CDC5L inhibits xenograft tumour growth *in vivo*. Tumour growth curve (A) and tumour sizes (B) after T24 cells stably expressing low levels CDC5L or empty vectors were injected into 4-week-old nude mice. (C) Fewer Ki-67+ cells and lower CDC5L expression levels were detected by IHC in sh-CDC5L treated tumours. (D) Proteins were extracted from the tumour xenografts. CDC5L, p-AKT, and AKT expression was detected by western blotting. β-actin was used as an internal control. *P<0.05, **P<0.01.

Furthermore, to explore the function of CDC5L *in vivo*, nude mouse experiments were performed after establishing stable sh-CDC5L cells. After 5 weeks of tumour implantation, the tumour xenograft sizes of the sh-CDC5L group were dramatically reduced compared with the sh-NC group (Fig. 3A). Consistently, the sh-CDC5L group showed slower growth of the tumour xenografts compared with the sh-NC group (Fig. 3B). In addition, we detected the expression of Ki67 in the two groups immunohistologically. As shown in Fig. 3C, the tumour tissues from the CDC5L knockdown group showed lower CDC5L expression levels and carried fewer Ki-67+ cells than in the control group, indicating a critical role for CDC5L in promoting bladder cancer growth *in vivo*.

To further explore the possible mechanism underlying the cell growth inhibition effect, we examined expression of PI3K/AKT family protein members by western blot. As illustrated in Fig. 3D, upon knockdown of CDC5L, the total phosphorylation level did not change but AKT phosphorylation was decreased. From the results above, we concluded that CDC5L plays a critical role in cell growth and proliferation partially through regulation of the PI3K/AKT signalling pathway.

### Knockdown of CDC5L promoted bladder cancer cell apoptosis

**Figure 4:**
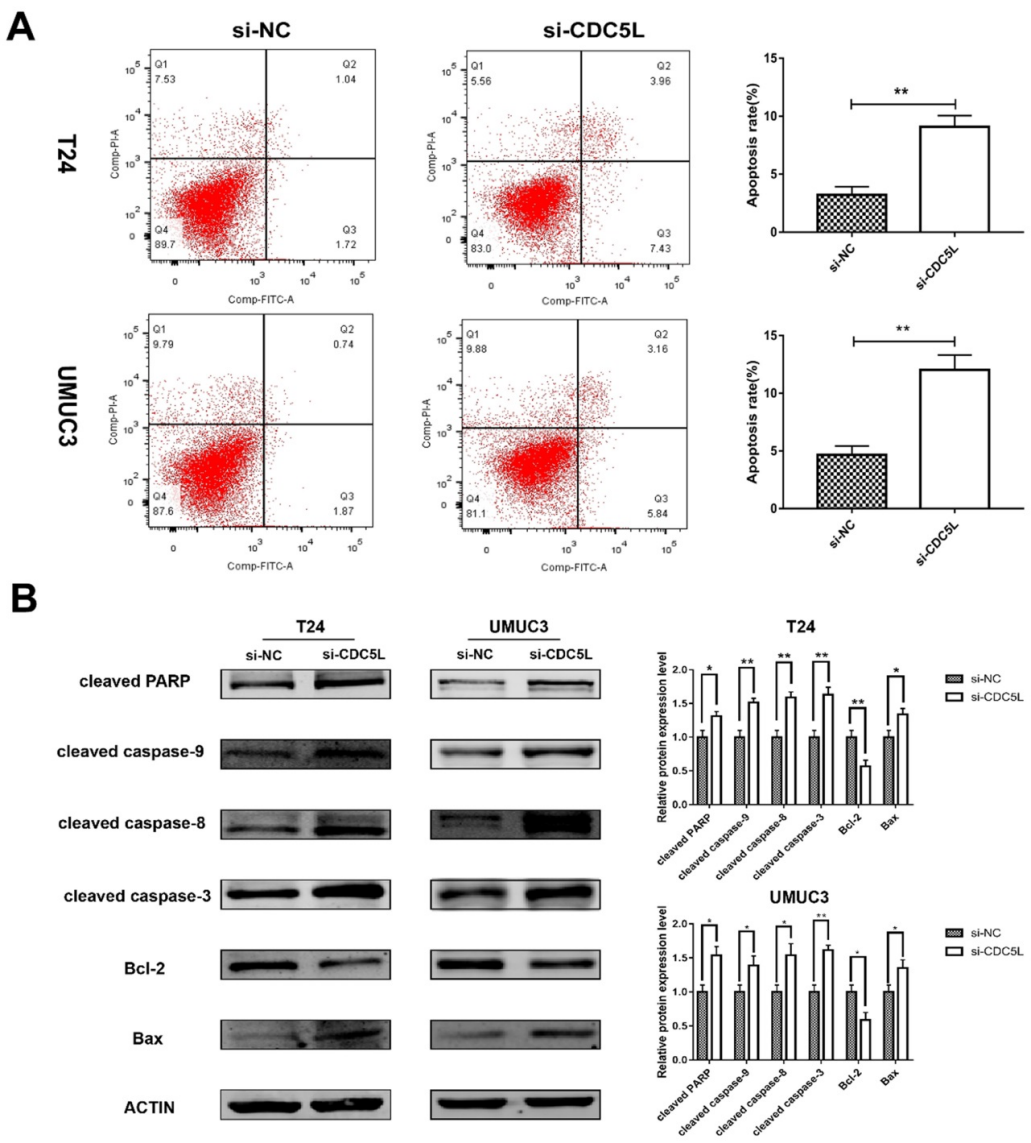
Reduction of CDC5L promoted bladder cancer cell apoptosis. (A) Flow cytometry assay for apoptotic rates in T24 and UMUC3 cells following CDC5L knockdown. (B)Representative cell apoptosis markers were determined by Western blot assay in T24 and UMUC3 cells, respectively. β-actin was used as an internal control. *P<0.05, **P<0.01 (n=3).

A previous study has shown that CDC5L is a regulator of mitotic progression 15, and so we predicted that CDC5L might have effects on bladder cancer cell apoptosis. Flow cytometry was used to analyse cell apoptosis. The percentage of cells at different phases revealed that CDC5L knockdown induced an increase in apoptosis of T24 and UMUC3 cells (Fig. 4A). To further understand the effect of CDC5L on bladder cancer cell apoptosis, the expression of several apoptosis related proteins was determined in bladder cancer cells after CDC5L knockdown. The protein expressions of the pro-apoptotic markers cleaved-PPAR, caspases 3, 8, and 9, and Bax were found to be upregulated while the protein expression of the anti-apoptotic marker Bcl-2 was found to be downregulated (Fig. 4B). Taken together, the current data indicated that CDC5L promotes the growth of bladder cancer due to the inhibition of apoptosis.

### Knockdown of CDC5L inhibits migration and invasion of bladder cancer cells

**Figure 5:**
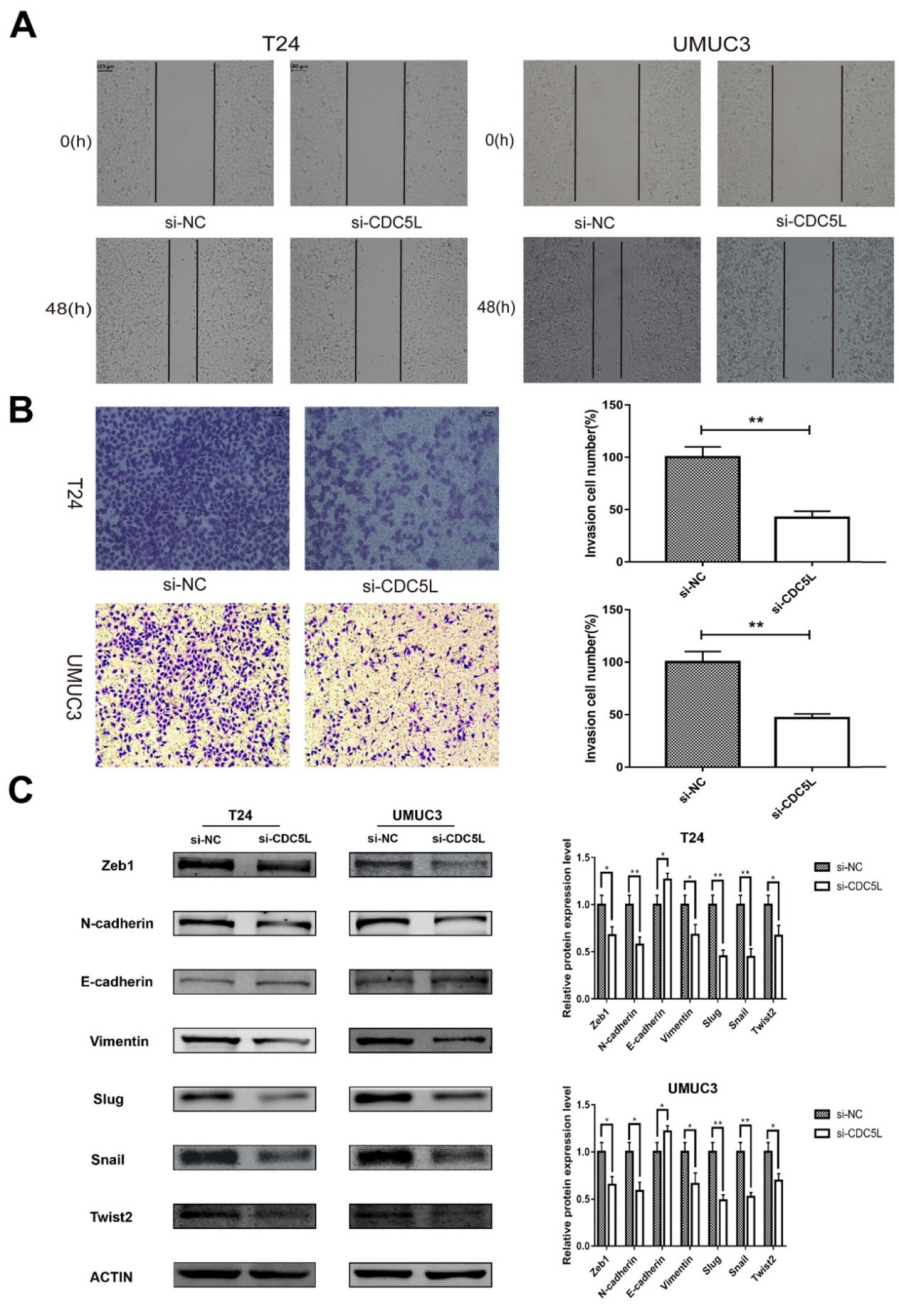
knockdown of CDC5L inhibits bladder cancer cell migration, invasion and EMT. Wound healing assay (A) and Transwell assay (B) were performed to examine migration and invasion of T24 and UMUC3 cells. (C) EMT-associated markers were measured by western blot analysis in T24 and UMUC3 cells, respectively. β-actin was used as an internal control. *P<0.05, **P<0.01 (n=3).

To investigate the effect of CDC5L on the metastatic and invasion capacity of bladder cancer cells, cell scratch assay and Transwell assay were performed to examine migration and invasion abilities of both T24 and UMUC3 cells, respectively. It was observed that knockdown of CDC5L significantly reduced the tumour cell migration and invasion abilities compared to the control group (Fig. 5A and 5B).

### Knockdown of CDC5L inhibits bladder cancer epithelial-mesenchymal transition (EMT)

EMT is a cellular program that is known to confer on cancer cells increased tumour-initiating and metastatic potential 19. The expression of several EMT- related markers was also affected by the knockdown of CDC5L. In this study, knockdown of CDC5L significantly reduced the protein expression of mesenchymal markers N-cadherin and vimentin as well as the transcription factors Zeb1, Slug, Snail and Twist2 compared to the si-NC group. Conversely, the expression level of the epithelial marker E-cadherin was significantly upregulated (Fig. 5C). Therefore, our results suggest CDC5L contributes to promotion of EMT in bladder cancer progression.

## Discussion

With the development of molecular biology, the application of genetic diagnostic techniques promises to change bladder cancer clinical practice in the future 20. Great progress has been made in the management of patients with bladder cancer over the past few years; however, the underlying molecular mechanisms of this disease remain to be studied. Here, we found that CDC5L was highly expressed in bladder cancer and played an important role in the tumorigenesis of bladder cancer.

Cell cycle regulation plays a critical role in malignant transformation and in the development of resistance to chemotherapy. Thus, targeting the cell cycle is increasingly considered an approach to cancer therapy 21, 22. A previous study has shown that CDC5L is a regulator of mitotic progression, as depletion of CDC5L inhibits mitotic progression and eventually leads to mitotic catastrophe 15. CDC5L was involved in the progression of hepatocellular carcinoma and was significantly associated with multiple clinicopathological factors. Depletion of CDC5L induced cell cycle arrest at the G2/M phase and reduced cell growth of hepatocellular carcinoma 23. CDC5L has a similar effect in gliomas. Downregulation of CDC5L results in the apoptosis of glioma cells 24. Therefore, CDC5L is related to cell size and cell proliferation. Amplification of chromosomal bands 6p12‐p21 appeared to be significant in the pathogenesis of osteosarcoma, and CDC5L is found to be the most likely candidate oncogene for the 6p12-p21 amplicon 25.CDC5L was highly expressed in colon cancer cells and was correlated with cell growth and proliferation partially by regulating the PI3K/AKT signalling pathway. Consistently, we found that depletion of CDC5L in bladder cancer cells induced a decrease in AKT phosphorylation, but the total phosphorylation level did not change.

**Figure 6:**
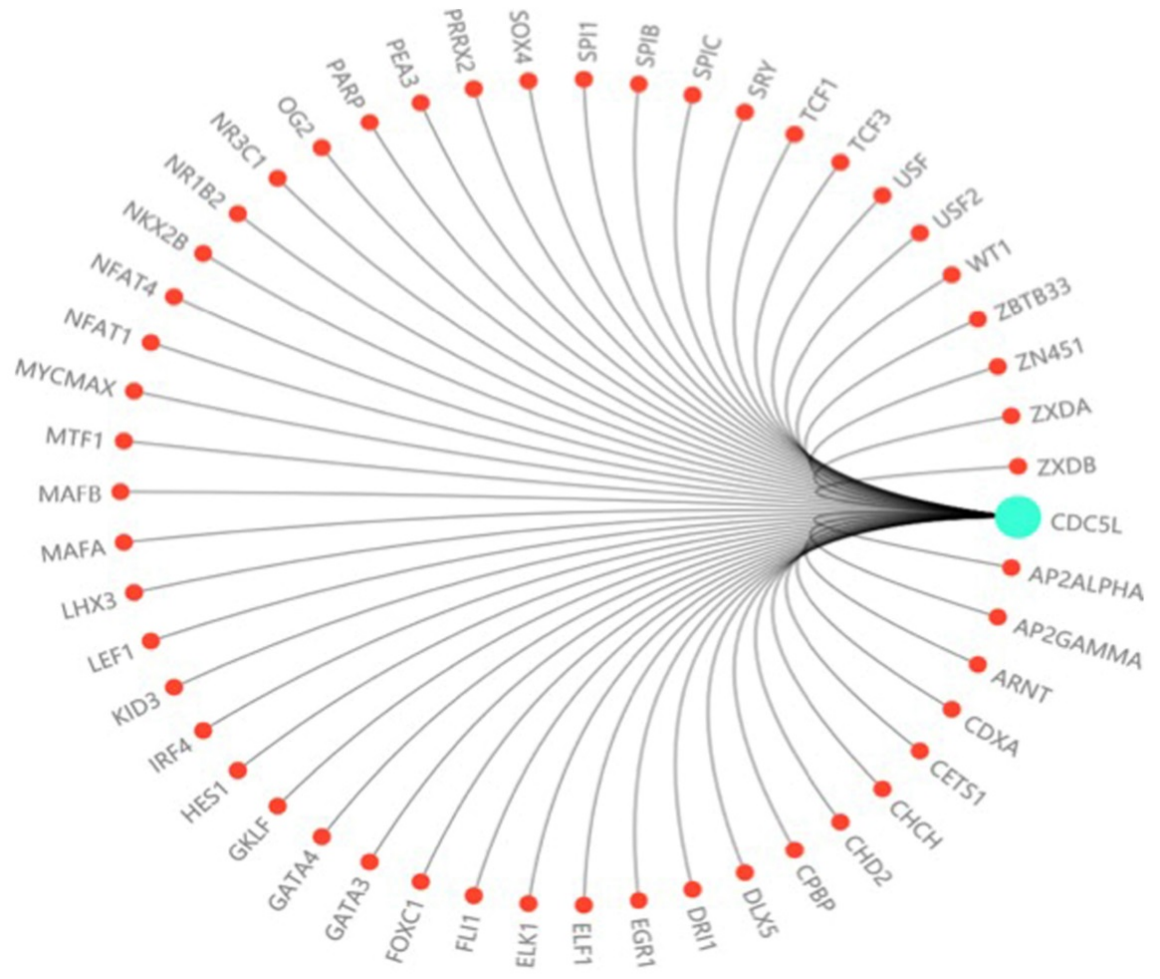
The prediction of transcription factor related to CDC5L. The highly recommended transcription factor related to CDC5L was obtained by integrating the information of the Transfac database, COSMIC database and dbSNP database.

In this study, we investigated the effect of CDC5L on bladder cancer. CDC5L was highly expressed in bladder cancer and the expression of CDC5L was associated with multiple clinicopathological factors. Knockdown of CDC5L inhibited the growth of bladder cancer by promoting cell apoptosis. CDC5L also promoted cell proliferation by regulating PI3K/AKT signalling. Animal model assays suggested that CDC5L promotes cell proliferation not only *in vitro* but also *in vivo*. And CDC5L has a positive effect on the tumour cell migration and invasion abilities by cell scratch assay and Transwell assay. We have further confirmed that CDC5L plays an important role in the metastasis of bladder cancer through the validation of clinical patient datas. In addition, we demonstrated for the first time that CDC5L has a significant effect on bladder cancer cell EMT, which closely relates to bladder cancer cell metastasis. Moreover, further study is needed to fully understand the underlying mechanism of CDC5L in the development of bladder cancer. For example, according to the predicted score of the Transfac database and the information of the COSMIC database and dbSNP database, we predicted dozens of highly recommended transcription factors related to CDC5L (Fig. 6). These transcription factors may regard as an important way to study the upstream signalling pathway of this gene.

## Conclusions

In summary, these findings suggest that CDC5L may play important roles in bladder cancer and we have confirmed that CDC5L contribute to the tumorigenesis of bladder cancer *in vitro* and *in vivo*. Therefore, CDC5L may serve as a novel target for bladder cancer therapy in the future.

## Figures and Tables

**Table 1:** Correlation of CDC5L expression with clinicopathological factors in 65 bladder cancer patients.

Characteristics	Total	CDC5L expression		*P* value
		Low	High	
Total	65	25	40	
Age (years)				0.136
≤65	34	16	18	
>65	31	9	22	
Gender				0.542
Male	59	22	37	
Female	6	3	3	
Smoking status				0.724
No	33	12	21	
Yes	32	13	19	
BMI				0.170
≤24	33	10	23	
>24	32	15	17	
Diabetes status				0.316
No	51	18	33	
Yes	14	7	7	
Histologic grade				0.023*
High	8	6	2	
Low	57	19	38	
Tumor stage				0.865
T1-T2	20	8	12	
T3-T4	45	17	28	
Lymphatic invasion				0.041*
Negative	45	21	24	
Positive	20	4	16	
Distant metastasis				0.047*
Negative	60	21	39	
Positive	5	4	1	

*p<0.05.
