# Depletion of CDC5L inhibits bladder cancer tumorigenesis: Erratum

**DOI:** 10.7150/jca.75714

**Published:** 2022-08-19

**Authors:** Ziwei Zhang, Weipu Mao, Longsheng Wang, Mengnan Liu, Wentao Zhang, Yuan Wu, Junfeng Zhang, Shiyu Mao, Jiang Geng, Xudong Yao

**Affiliations:** Department of Urology, Shanghai Tenth People's Hospital, Tongji University, Shanghai 200072, P. R. China.

In our paper, the Western Blot bands #01 and #03 in Figure 1C were reused due to an error in editing the pictures, and the same error occurs in Figure 2B and Figure 2D. So we replace the correct picture in Figure 1C, Figure 2B and Figure 2D. We are deeply sorry and sincerely apologize for the errors and for any inconvenience that may cause to the readers and the editors of this journal. Figure 1C, Figure 2B and Figure 2D was corrected as follows.

## Figures and Tables

**Figure 1:**
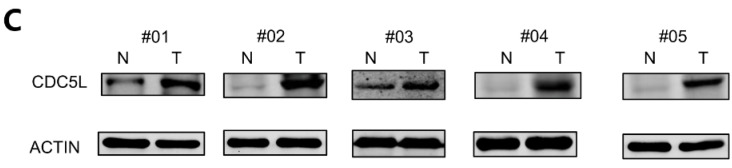
(C) Protein levels of CDC5L in bladder cancer tissues (T) and paired adjacent normal tissues (N) detected by western blot.

**Figure 2:**
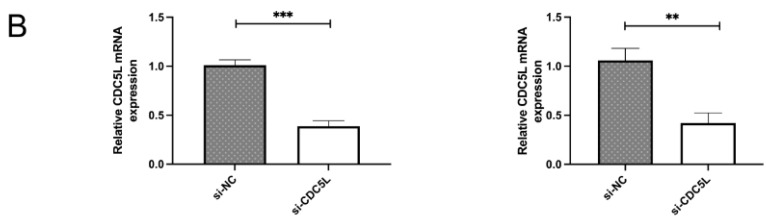

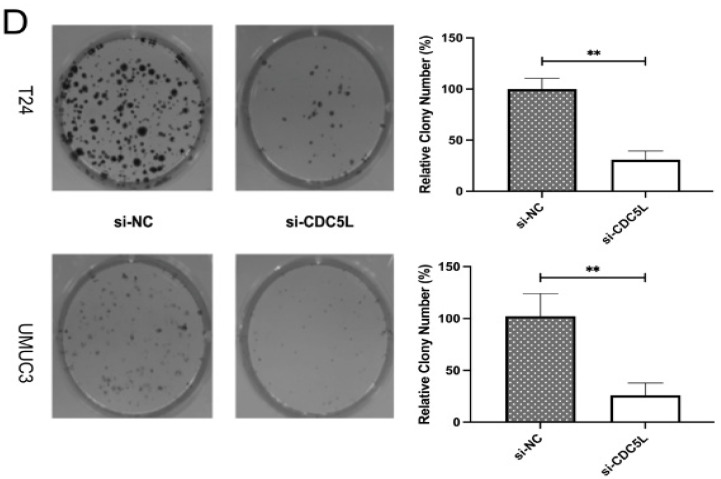
**(B)** CDC5L mRNA expression was detected by qRT-PCR 48h after transfection of si-CDC5L or si-NC. **P<0.01, ***P<0.001 (n=3). Colony formation assay **(D)** were used to determine proliferation and colony-forming ability of si-CDC5L or si-NC-transfected T24 and UMUC3 cells. **P<0.01 (n=3).

